# Understanding the impact of psychosocial working conditions on workers’ health: we have come a long way, but are we there yet?

**DOI:** 10.5271/sjweh.3984

**Published:** 2021-09-30

**Authors:** Ida EH Madsen, Reiner Rugulies

**Affiliations:** National Research Centre for the Working Environment, Copenhagen, Denmark; Department of Public Health and Department of Psychology, University of Copenhagen, Denmark

This issue of the journal includes a meta-review, ie, a systematic review of systematic reviews, summarizing the published evidence on the associations between exposure to adverse psychosocial working conditions and risk of developing diseases or disorders during the past 20 years (1). Although the authors allowed inclusion of reviews reporting results from cross-sectional studies, the majority of the included reviews were restricted to prospective cohort studies – the gold standard method in psychosocial occupational epidemiology. We commend the authors for their succinct summary of the current knowledge on the topic, encompassing this multitude of exposures and outcomes in one single paper. The paper finds that there is consistent evidence of associations between certain psychosocial working conditions (job strain, effort-reward imbalance, job insecurity, long working hours) and certain health conditions (cardiovascular diseases and mental disorders, in particular depression). The paper also identifies the lack of studies concerning numerous other working and health conditions, as elegantly depicted in their figure 1, showing the presence or absence of reviews concerning all combinations of the included exposures and outcomes.

## The early days of psychosocial occupational epidemiology

Compared to other fields of occupational health, research on psychosocial working conditions and health is a relatively recent discipline (2). One of the first studies on the topic was a paper by Friedman et al, published in 1958, reporting increased cholesterol levels and reduced blood clotting time among tax accountants during a period of putative high occupational stress, the annual April 15^th^ tax filing deadline in the United States (3). Curiously, though, this observation did not inspire research on occupational stressors but rather led to the development of the concept of “type A behavior”, a behavioral pattern characterized by feelings of time urgency, competitiveness and hostility that became the dominant psychosocial explanation for risk of coronary heart disease in the late 1970s and early 1980s (4). The concept later largely disappeared from the discussion as findings from earlier epidemiological studies could not be replicated (5). In Belgium, Kornitzer and colleagues published a paper in 1975 on the risk of coronary heart disease in employees at two banks, and discussed whether the higher occurrence in one of the banks could be related to work organization (6), a hypothesis which they later examined and corroborated (7). In the 1960s in Sweden, Gardell, Frankenhaeuser and others pioneered both theoretical concepts and empirical research on the role of work under- and overload, participation and alienation for both workplace democracy and workers’ health (8-10). This research inspired, among other things, the development of the demand–control model (job strain model) (11) that was tested in Swedish cohorts from the early 1980s (12, 13). The demand-control model quickly became the dominant approach for understanding the contribution of psychosocial working conditions to risk of cardiovascular disease, but reviews of these studies showed inconsistent results (14, 15). A major advance was made in 2012, when the “Individual-Participant Data Meta-Analysis in Working Populations (IPD-Work) Consortium published pooled estimates from 13 European cohort studies with almost 200 000 participants, showing a prospective association between exposure to job strain and risk of coronary heart disease (16). A key novelty of the IPD approach was to apply harmonized measures of exposures and outcomes in all included cohorts. Subsequent papers from the IPD-Work consortium showed associations between job strain and stroke (17), diabetes (18) and depression (19), between long working hours and coronary heart disease and stroke (20), diabetes (21) and depression (22) and between effort–reward imbalance and coronary heart disease (23).

Whereas research on psychosocial work environment and risk of cardiovascular disease has a long history, dating back to the 1980s, research on psychosocial work environment and mental disorders emerged only towards the end of the 1990s, but then rapidly accelerated. When Stansfeld & Candy published the first systematic review and meta-analysis on psychosocial working conditions and common mental disorders in this journal in 2006, they identified only 11 papers (24). In contrast, a recent review by Mikkelsen et al identified 56 papers on the association between psychosocial working conditions and risk of incident clinical depressive disorders (25).

## The past 20 years of research

The meta-review by Niedhammer et al only included reviews with meta-analyses that were published between 2000 and 2020. Given the acceleration of research and the growing number of studies published on the topic, this is a reasonable approach to provide an overview of the current knowledge base. Despite the restriction to the last 20 years, Niedhammer et al identified no less than 72 eligible review studies, a clear indicator of the massive proliferation of studies and the maturation of the research field.

Given this vast number of studies, it is timely to ponder what we have learned. For outcomes such as cardiovascular diseases and depression, the included reviews show rather consistently that employees who report certain psychosocial working conditions, in particular job strain, effort–reward imbalance, job insecurity and long working hours, are at increased risk. But how certain can we be that these associations are causal? First, caution is needed because most of the pooled estimates are modest, usually <2.0 and often <1.5. In the presence of numerous other well-established risk factors, such modest risk estimates make residual confounding a crucial issue. This discussion about causality is not new, and many arguments, such as those related to possible bias due to self-reported data, were raised decades ago (26, 27). Despite the massive research efforts, as evident by the number of studies published, it seems some disputes remain unchanged. For example, the above-mentioned recent review by Mikkelsen et al reported numerous associations between psychosocial working conditions and risk of depressive disorders (25), confirming and extending the results of the meta-review (1). However, due to methodological limitations of the literature, the authors did not feel confident to conclude whether psychosocial working conditions are likely or unlikely to cause depressive disorders.

## So what’s next?

So how can we move the research field of psychosocial working conditions and health forward? The discussion of causal inference, and how to arrive at it, is not limited to occupational health research. It is a topic of intense debate amongst epidemiologists and philosophers alike, and various approaches exist to establishing causality (28). While some have argued that applying well-defined hypotheses that correspond to potential interventions in combination with certain statistical methods and a counterfactual framework may lead to causal inference (29), others have argued that this approach is overly restrictive and risks limiting the topics that can be researched and the types of evidence that can be considered (30). The latter group proposes that causal claims are arrived at by piecing together bits of evidence from diverse studies, each with their own inherent strengths and weaknesses. Together these studies form a broader picture, like pieces of a puzzle, based on which we can form our judgement. Each study contributes only part of the whole and must be considered in light of the extant knowledge, with a keen eye on ruling out alternative hypotheses.

With this in mind, we propose that the identification of alternative hypotheses – in order to rule them out – may be an important next step. Much criticism of psychosocial work environment research has focused on the role of potential biases related to the self-reported nature of exposure measurements in most studies on psychosocial working conditions and health, and calls have been made for studies measuring exposures objectively (26, 27). While the term objective may certainly also be debated (26), we and other research groups have been making steps to meet this challenge by applying non-self-reported exposure measures (31, 32), work unit aggregations (33, 34) or job exposure matrices to measure working conditions (35–37). These measures also have their limitations. Job exposure matrices, for example, are vulnerable to non-differential misclassification, issues related to validation, and are unable to measure day-to-day or between-worker variation within the assigned occupational grouping (38). Consequently these studies should also be seen as only small pieces of the bigger puzzle. But within these limits, they may be considered small steps to rule out the alternative hypothesis of confounding due to reporting bias. Other small steps may be fixed-effects analyses examining intra-individual changes and thereby controlling for time-invariant confounders (39) or studies that analyze the association between onset of exposure and subsequent incident health outcomes (40). Alternative hypotheses may also pertain to the possibility of residual confounding due to factors such as personality, genetics, or life events outside the workplace (41–43). Ruling out these alternative hypotheses – and identifying more – could be considered important next steps for the research field.

The issue of causality is not only a technical and somewhat academic discussion. From the viewpoint of those many individuals who believe that they have acquired a health problem due to their psychosocial working conditions, the consequences of this rather academic discussion are very real. More evidence for a causal relationship could result in changes to compensation practices, which would make a tangible difference in the lives of these individuals. At the workplace and societal level, more certainty concerning causality could motivate preventive practices and possibly help prevent the potential adverse health consequences of psychosocial working conditions before they occur – a valuable goal for any public health professional, academic or not.

## Conflicts of interest

The two authors are members of the IPD-Work Consortium and have been involved in several of the reviews that were included in the meta-review.
